# Renin–angiotensin imbalance promotes excessive fibrin deposition through M2 macrophage–derived tissue factor expression in eosinophilic chronic rhinosinusitis

**DOI:** 10.1007/s00011-026-02254-1

**Published:** 2026-04-28

**Authors:** Tetsuji Takabayashi, Kanako Yoshida, Yukinori Kato, Masafumi Sakashita, Shigeharu Fujieda

**Affiliations:** The Division of Otorhinolaryngology Head and Neck Surgery, Department of Sensory and Locomotor Medicine, University of Fukui, 23 Shimoaizuki, Matsuoka, Yoshida, Fukui 910-1193 Japan

**Keywords:** Nasal polyps, M2 macrophage, Tissue factor, Fibrin, Eosinophilic chronic rhinosinusitis

## Abstract

**Background:**

Recalcitrant nasal polyps are hallmarks of eosinophilic chronic rhinosinusitis, with abnormal fibrin deposition being critical for nasal polyp development. However, the specific cellular and molecular mechanisms underlying the pathogenesis of nasal polyps in patients with eosinophilic chronic rhinosinusitis remain poorly understood. To assess the impact of the renin-angiotensin system on eosinophilic chronic rhinosinusitis pathogenesis and identify new therapeutic targets.

**Methods:**

Renin-angiotensin system components, fibrin, tissue factor, and macrophages in nasal tissues were assessed using real-time polymerase chain reaction, enzyme-linked immunosorbent assay, immunohistochemistry, and immunofluorescence. Gene expression and protein levels were also analyzed in cultured THP-1 cells.

**Results:**

In eosinophilic chronic rhinosinusitis nasal polyps, angiotensin-converting enzyme 2, Mas receptor, and angiotensin (1–7) were significantly reduced, while angiotensin II receptor type 1 was increased compared to non-eosinophilic chronic rhinosinusitis nasal polyps. This suggests angiotensin II/angiotensin II receptor type 1 axis predominance over the angiotensin (1–7)/Mas receptor axis in eosinophilic chronic rhinosinusitis. Immunofluorescence revealed profound fibrin deposition and increased tissue factor levels in eosinophilic chronic rhinosinusitis nasal polyps. M2 macrophages (CD68 + /CD163 +) were highly infiltrated, with most expressing tissue factor. Tissue factor expression was significantly increased in M2-polarized macrophages, and co-stimulation with angiotensin II further enhanced this expression.

**Conclusion:**

Renin-angiotensin system dysregulation might contribute to nasal polyp development in patients with eosinophilic chronic rhinosinusitis. Angiotensin II/angiotensin II receptor type 1 axis predominance over the angiotensin (1–7)/Mas receptor axis may enhance fibrin deposition by enhancing tissue factor expression in M2 macrophages, contributing to recalcitrant nasal polyp formation.

**Supplementary Information:**

The online version contains supplementary material available at 10.1007/s00011-026-02254-1.

## Introduction

Chronic rhinosinusitis (CRS) is one of the most common diseases worldwide and classifying it according to inflammatory endotypes can enhance the understanding of its underlying pathophysiology. CRS is divided into eosinophilic (ECRS) and non-eosinophilic (non-ECRS) subtypes. ECRS, characterized by type 2 inflammation, is usually more refractory and severely symptomatic than non-ECRS [1]. The primary targets for treating ECRS are nasal polyps (NPs) or polypoid sinus mucosa, causing persistent nasal congestion, olfactory disturbances, facial pain, and headaches. However, the exact mechanism underlying NP formation remains unclear.

The renin-angiotensin system (RAS) is a critical hormonal regulator of blood pressure and hydroelectrolyte balance [2]. Renin induces angiotensin I production, which is converted to the vasoconstrictive peptide angiotensin II by angiotensin-converting enzyme (ACE). Angiotensin II exerts its biological functions through two G-protein-coupled receptors: angiotensin II receptor type 1 (AT1R) and angiotensin II receptor type 2. Angiotensin-converting enzyme 2 (ACE2) is a homolog of ACE that counterbalances ACE activity by converting angiotensin II to angiotensin-(1–7) (Ang-(1–7)), which binds to the Mas receptor (MASR).

Conventionally, RAS is viewed as a systemic regulatory mechanism; however, recent studies have demonstrated that RAS also plays a critical role in various organs, and its dysregulation is implicated in various diseases [3–5].

Severe acute respiratory syndrome coronavirus 2 (SARS-CoV-2), the virus responsible for coronavirus disease 2019 (COVID-19), enters human respiratory cells by binding to ACE2 [6]. This interaction significantly downregulates ACE2 receptor activity, promoting RAS dysregulation. Histopathologically, in patients with poor COVID-19 prognosis, the lungs exhibit intense fibrin deposition in the alveolar and interstitial spaces, driven by coagulation activation and coincident fibrinolysis inhibition [7, 8].

Similar excessive fibrin deposition in NP tissues has been reported previously. This excessive fibrin deposition likely contributes to the retention of exuded plasma proteins from capillaries due to severe allergic inflammation, prolonging mucosal edema and playing an etiological role in NP formation [9]. Nattokinase, a serine protease with strong fibrinolytic activity, effectively shrinks surgically obtained NP tissue through fibrin degradation [10]. Subsequent studies have revealed that type 2 inflammation enhances coagulation and diminishes fibrinolysis, resulting in excessive fibrin deposition in nasal mucosa [9, 11, 12].

Recent studies have also reported decreased ACE2 levels in airway type 2 inflammatory diseases, including CRS and asthma [13–15]. Therefore, we hypothesized that RAS dysregulation contributes to excessive fibrin deposition, promoting recalcitrant NP formation. To assess this, we investigated the expression of RAS components in the sinonasal tissue of patients with CRS, specifically examining RAS dysregulation involvement in NP development in patients with ECRS.

## Materials and methods

### Patients and sample preparation

Patients with CRS with NP (CRSwNP) were recruited from the Department of Otorhinolaryngology, Head and Neck Surgery at the University of Fukui. NP tissues were obtained from these patients during routine functional endoscopic sinus surgery. All patients met the criteria for CRS as defined by the guidelines of the European position paper on rhinosinusitis and nasal polyps [16]. Table E1presents the patients’ characteristics. Patients with > 70 eosinophils per high-power field in their NP specimens were classified as having ECRS [17]. All patients provided informed consent, and the study protocol was approved by the institutional review board of the University of Fukui, in accordance with the ethical principles contained in the Declaration of Helsinki. Further details are provided in the online supplement.

### Real-time polymerase chain reaction (PCR)

Nasal tissues were stabilized in RNAlater (Thermo Fisher Scientific, Waltham, MA, USA) and homogenized using a Multi-Beads Shocker (Yasui Kikai Corporation, Osaka, Japan), according to the manufacturer’s instructions. Total RNA was extracted using NucleoSpin RNA II (Macherey–Nagel, Bethlehem, PA, USA) with DNase I (Invitrogen, Carlsbad, CA). Single-stranded cDNA was synthesized using the High-Capacity cDNA Reverse Transcription Kit (Thermo Fisher Scientific). Semi-quantitative real-time PCR was performed using the TaqMan method on an Applied Biosystems StepOnePlus Real-Time PCR system (Thermo Fisher Scientific). Further details are provided in the online supplement.

### Immunohistochemistry

Blocked sections were incubated overnight at 4 °C with mouse anti-human ACE2 mAb (666991-Ig; Proteintech, Rosemont, IL), mouse anti-human MAS1 mAB (sc-390453; Santa Cruz Biotechnology, Dallas, TX), rabbit anti-human AGTR1/AT1R Ab (LS-C490145; Lynnwood, WA), mouse anti-human fibrin mAb (SEKISUI diagnostics, Stamford, CT), rabbit anti-human tissue factor antibody (bs-4690R; Bioss Antibodies, Woburn, MA), mouse anti-human CD68 mAb (Thermo Fisher Scientific), or mouse anti-human CD163 mAb (Thermo Fisher Scientific). Further details are provided in the online supplement.

### Enzyme-linked immunosorbent assay (ELISA)

Angiotensin II (Montigny-le-Bretonneux, France), Ang-(1–7) (Cloud-Clone Corp., Wuhan, China), and tissue factor (TF) (R&D Systems, Minneapolis, MN, USA) levels were assayed using specific ELISA kits according to the manufacturer’s instructions. Further details are provided in the online supplement.

### Cell culture

THP-1 cell culture methods are detailed in the online supplement.

### Statistical analysis

All data are reported as mean ± SEM unless otherwise noted. Between-group differences were analyzed using the Kruskal–Wallis analysis of variance with Dunnett’s post-hoc test or the Mann–Whitney U test, as appropriate. Correlations were assessed using Spearman’s rank correlation. A *P*-value < 0.05 was considered statistically significant.

## Results

### ACE and ACE2 expression in patients with CRS

**Fig. 1:**
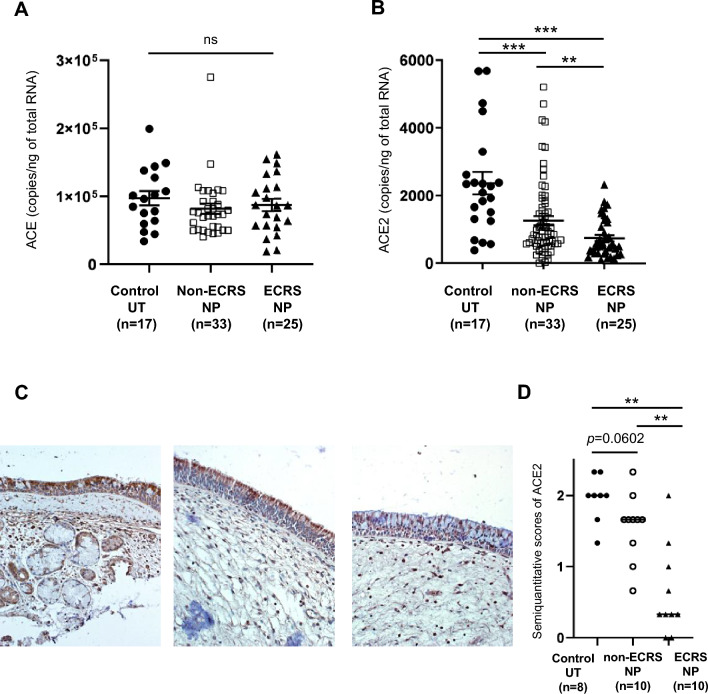
Expression of angiotensin-converting enzyme (ACE) and ACE2 in nasal tissues. Total RNA was extracted from uncinate tissue (UT) and nasal polyps (NPs) of patients with eosinophilic chronic rhinosinusitis (ECRS) and non-ECRS, and expression levels of ACE (A) and ACE2 (B) were analyzed using real-time polymerase chain reaction (PCR). Immunohistochemistry for ACE2 was performed using an anti-human ACE2 antibody. Representative images showing ACE2 immunostaining in the UT of a control subject (left panel), NP from a patient with non-ECRS (middle panel), and NP from a patient with ECRS (right panel) (C). (D) Semi-quantitative analysis of ACE2 expression in UT from control participants (n = 8), NPs from patients with non-ECRS (n = 10), and NPs from patients with ECRS (n = 10) was performed. Magnification: × 400. ** *P* < 0.01

Sinonasal and NP tissues were collected from 83 participants with non-ECRS, 95 participants with ECRS, and 30 control participants to determine RAS component levels, fibrin, TF, and macrophages in the nasal tissues of patients with CRS. Table E1lists the participants’ characteristics. We assessed ACE and ACE2 expression in NP tissues from patients with non-ECRS or ECRS, as well as in uncinate process tissues (UT) from control participants. Although ACE mRNA expression was not significantly different among the three groups (Fig. 1A), ACE2 mRNA levels significantly decreased in NP tissues from patients with ECRS than those from patients with non-ECRS and UT from control participants (Fig. 1B). To further characterize the ACE2 expression in patients with CRS, we performed immunohistochemical analysis of surgical samples from control, patients with non-ECRS, and patients with ECRS to determine whether ACE2 expression could be detected. We detected ACE2 staining primarily in the mucosal epithelium (Fig. 1C). Consistent with quantitative PCR data, ACE2 staining in the mucosal epithelium of NP tissues from patients with ECRS was less intense than that observed in control UT (Fig. 1C). Mucosal staining was graded for intensity by a blinded observer, as described in the online supplement. This semi-quantitative analysis showed significantly less ACE2 staining in the ECRS NP tissue than in the control UT tissue (Fig. 1D).

### Angiotensin levels in CRS

**Fig. 2:**
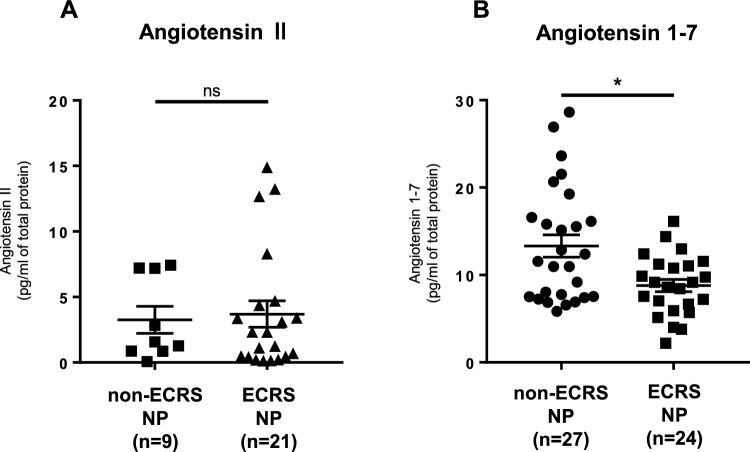
Levels of angiotensin II and angiotensin (1–7). Measurement of angiotensin II **A** and angiotensin (1–7) **B** in tissue homogenates of nasal polyps (NPs) tissues from patients with eosinophilic chronic rhinosinusitis (ECRS) and non-ECRS using ELISA. Concentrations were normalized to total protein levels. *P < 0.05

ACE2 counterbalances ACE activity, which promotes tissue inflammation and coagulopathy by converting angiotensin II into Ang-(1–7). Therefore, to determine the effects of decreased ACE2 levels on ECRS pathogenesis, we measured angiotensin II and Ang-(1–7) levels in nasal mucosa. Angiotensin II levels were considerably higher in NP tissues from patients with ECRS than in those from patients with non-ECRS, although the difference was not statistically significant (Fig. 2, left panel). Conversely, Ang-(1–7) levels were significantly lower in NP tissues from patients with ECRS than in those from patients with non-ECRS (Fig. 2, right panel). Collectively, these findings suggest that the imbalance between Angiotensin II and Ang-(1–7) may be caused by a reduction in ACE2 expression in NP tissues from patients with ECRS.

### AT1R and MASR expression in CRS

**Fig. 3:**
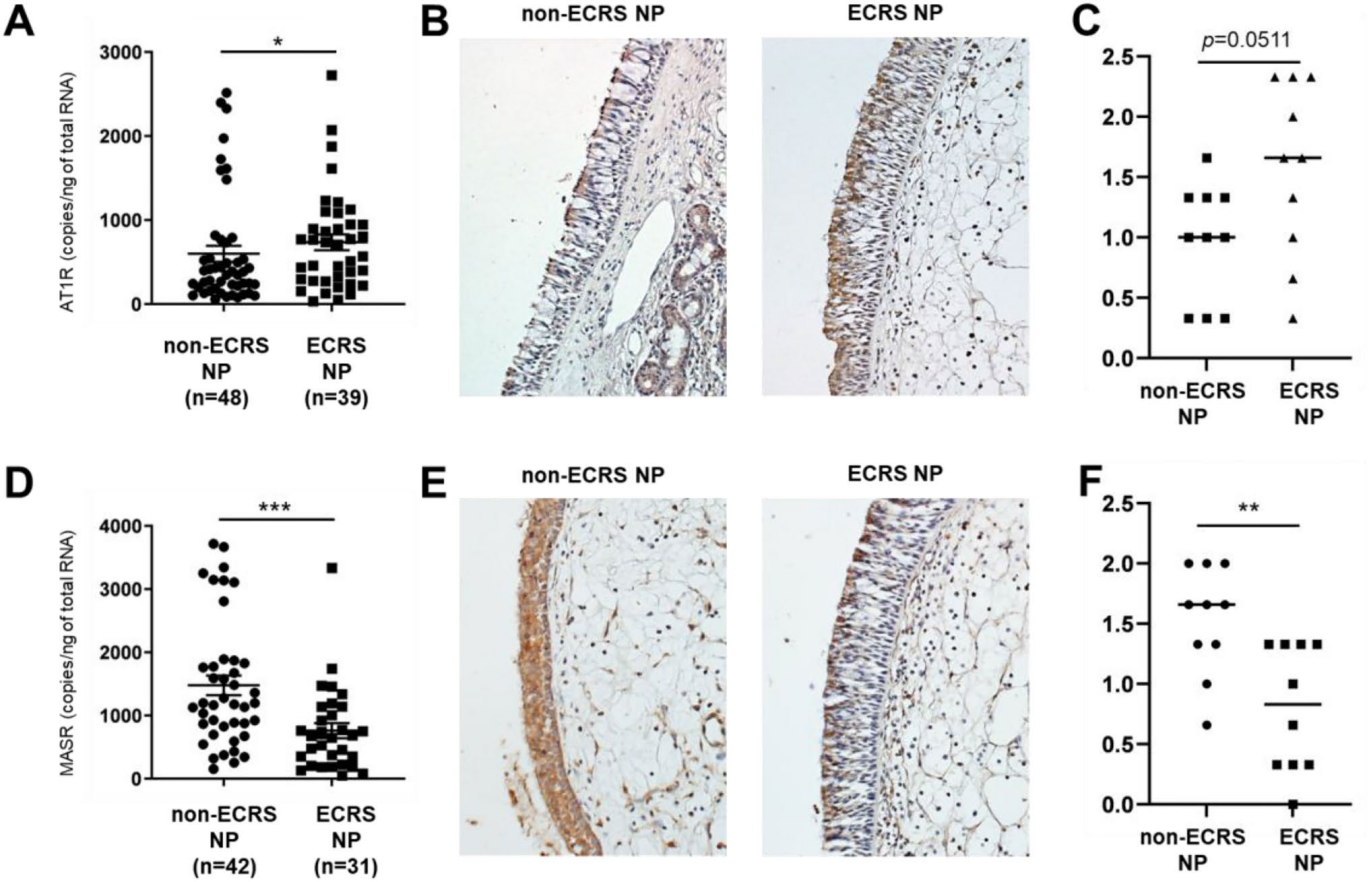
Expression of angiotensin II receptor type 1 (AT1R) and Mas receptor (MASR) in nasal polyps (NPs) tissues. Total RNA was extracted from NPs in patients with eosinophilic chronic rhinosinusitis (ECRS) and non-ECRS, and expressions of AT1R (**A**) and MASR (**D**) were analyzed using real-time PCR. Immunohistochemistry of angiotensin-converting enzyme 2 (ACE2) was performed with anti-human ACE2 antibody. Representative images showing immunostaining for AT1R in NP tissue from a patient with non-ECRS (**B**, left panel) and ECRS (**B**, right panel) and staining for MASR in NP tissue from a patient with non-ECRS (**E**, left panel) and ECRS (**E**, right panel). Semiquantitative analysis of ATIR (**C**) and MASR (**F**) expression in NPs from patients with non-ECRS (n = 10) and NPs from patients with ECRS (n = 10) was performed. Magnification: × 400. ** *P* < 0.01

We further analyzed the expression of AT1R and MASR, the main receptors for angiotensin II and Ang-(1–7), respectively, in the nasal mucosa of patients with CRS. We assessed AT1R and MASR expression in NP tissues from patients with non-ECRS and patients with ECRS using real-time PCR. AT1R mRNA levels were significantly higher in ECRS NP tissues (*P* < 0.05) than in non-ECRS NP tissues (Fig. 3A). Immunohistochemical analysis showed AT1R staining mainly in the mucosal epithelium and infiltrated cells in NP tissues. In agreement with the mRNA data, AT1R staining was higher in the ECRS NP than in the non-ECRS NP (Fig. 3B). Semi-quantitative analysis showed a trend toward increased AT1R expression in ECRS NP compared to non-ECRS NP. (*P* = 0.0511, Fig. 3C). Contrastingly, MASR mRNA levels were significantly lower in ECRS NPs (*P* < 0.001) than in non-ECRS NPs (Fig. 3D). Immunohistochemical analysis revealed MASR staining, primarily in the mucosal epithelium and infiltrated cells in NP. Consistent with the mRNA data, MASR staining was lower in ECRS NP than in non-ECRS NP (Fig. 3E). Semi-quantitative analysis revealed significantly less MASR staining in ECRS NPs than in non-ECRS NPs (Fig. 3F). Collectively, these findings suggest that the angiotensin II/AT1R axis is more dominant than the Ang-(1–7)/MASR axis in ECRS NPs. Reportedly, the angiotensin II/AT1R axis triggers tissue inflammation and the coagulation cascade, and the Ang-(1–7)/MASR axis counterbalances this, leading to decreased pathophysiological effects on tissues [18]. Therefore, we speculated that the angiotensin II/AT1R axis predominance in the nasal mucosa is involved in ECRS pathogenesis.

### Fibrin deposition and TF expression in CRS

**Fig. 4:**
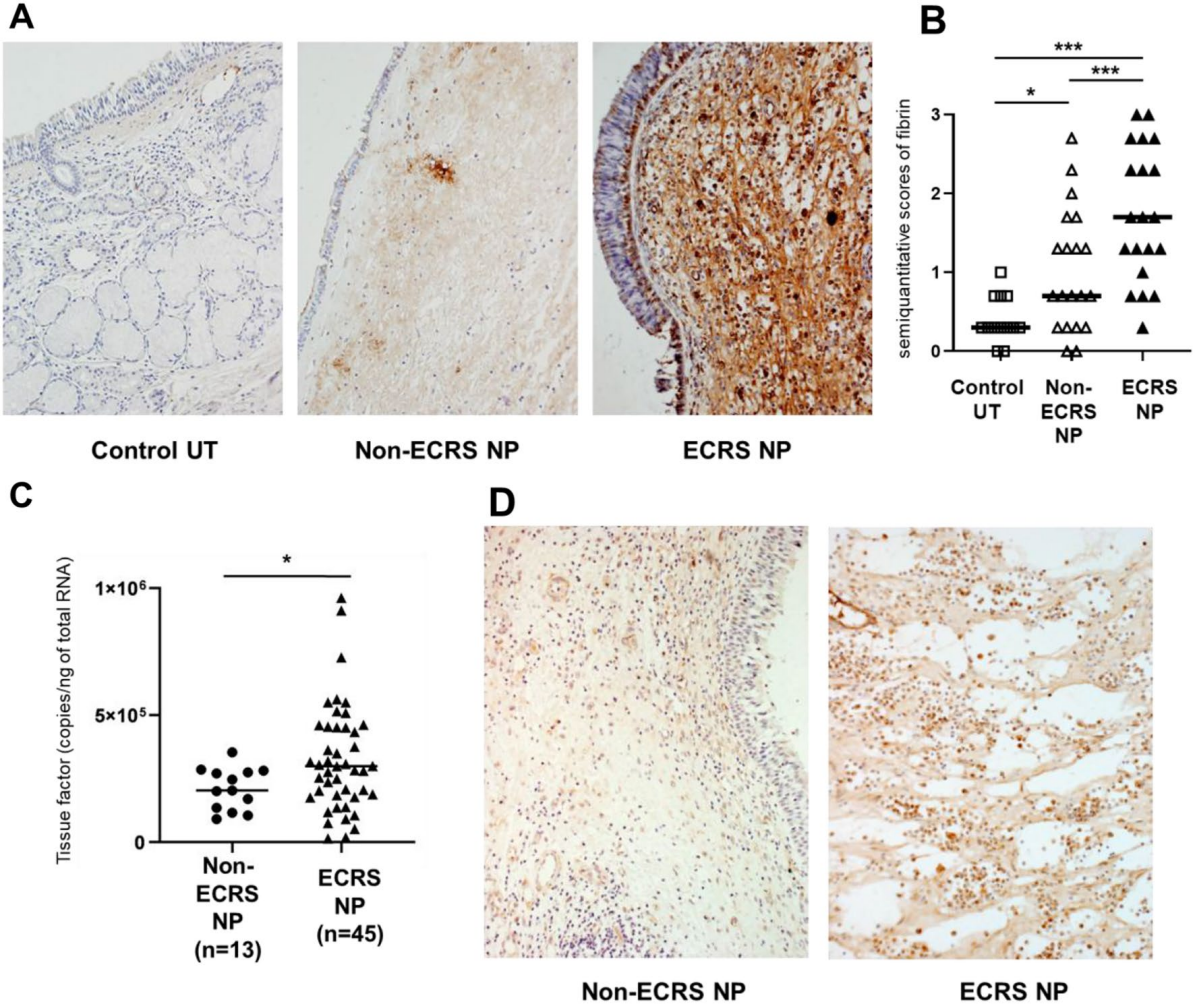
Fibrin deposition and tissue factor (TF) expression in nasal polyps (NPs) tissues. Immunohistochemical analysis of fibrin in NP tissue using anti-human fibrin antibody was performed. Representative images showing immunostaining for fibrin in the UT of a control participant (**A**, left panel), NP tissue from a patient with non-ECRS (**A**, middle panel), and ECRS (**A**, right panel). **B** Semiquantitative analysis of fibrin was performed on UT from control participants (n = 13) and NPs from patients with non-ECRS (n = 20) (**B**). Total RNA was extracted from NPs in patients with non-ECRS and ECRS, and the expression of tissue factor (TF) was analyzed using real-time PCR (**C**). Immunohistochemical analysis of TF was performed using an anti-human TF antibody. Representative immunostaining for TF in NP tissue from a patient with non-ECRS (**D**, left panel) and ECRS (**D**, right panel)

In patients with severe COVID-19, decreased ACE2 levels promote angiotensin II upregulation, which induces coagulation and inflammatory response, resulting in significant fibrin deposition in the lungs [8, 19]. We previously reported excessive fibrin deposition in NP tissues from patients with CRSwNP [9]. Excessive fibrin deposition is thought to contribute to the retention of exuded plasma proteins from capillaries, thereby prolonging mucosal edema and resulting in NP development. Thus, we sought to determine the differences in fibrin deposition in NPs between patients with non-ECRS and ECRS. To evaluate fibrin deposition in NP tissue, we performed immunohistochemical analysis of surgical samples from control participants and patients with non-ECRS and ECRS. Minimal fibrin staining was observed in the UT of control participants, while moderate levels were observed in the NP tissues of patients with non-ECRS (Fig. 4A). Contrastingly, intense fibrin staining was present in the submucosa of the NP tissues from patients with ECRS (Fig. 4A). Semi-quantitative analysis revealed significantly more intense fibrin staining in NP tissues from patients with ECRS compared with staining seen in UT from control participants or NP tissues from patients with non-ECRS (Fig. 4B). The coagulation cascade in inflammatory tissue is primarily TF-mediated, which triggers the extrinsic coagulation cascade and subsequent fibrin deposition in tissue [20]. Various cytokines induce TF expression and activation. The angiotensin II/AT1R axis also reportedly upregulates TF synthesis in monocytes, macrophages, vascular smooth muscle cells, and vascular endothelial cells [21, 22]. We next assessed TF expression in NP tissues from patients with non-ECRS and ECRS using real-time PCR. TF mRNA levels were significantly increased in ECRS NP tissues than in non-ECRS NP tissues (Fig. 4C). To further characterize TF protein expression in patients with ECRS, we performed immunohistochemical analysis of NP tissues from patients with non-ECRS and ECRS to determine whether TF expression could be detected. TF staining was detected primarily in submucosal inflammatory cells. Quantitative analysis demonstrated a significant increase in TF-positive cells in ECRS NP tissues compared with non-ECRS NP tissues (median 86.1 [n = 16] vs. 32.0 [n = 13] cells per high-power field, Mann–Whitney U test, two-tailed exact *P* < 0.0001) (Fig. 4D).

### Detection of TF in M2 macrophages

**Fig. 5:**
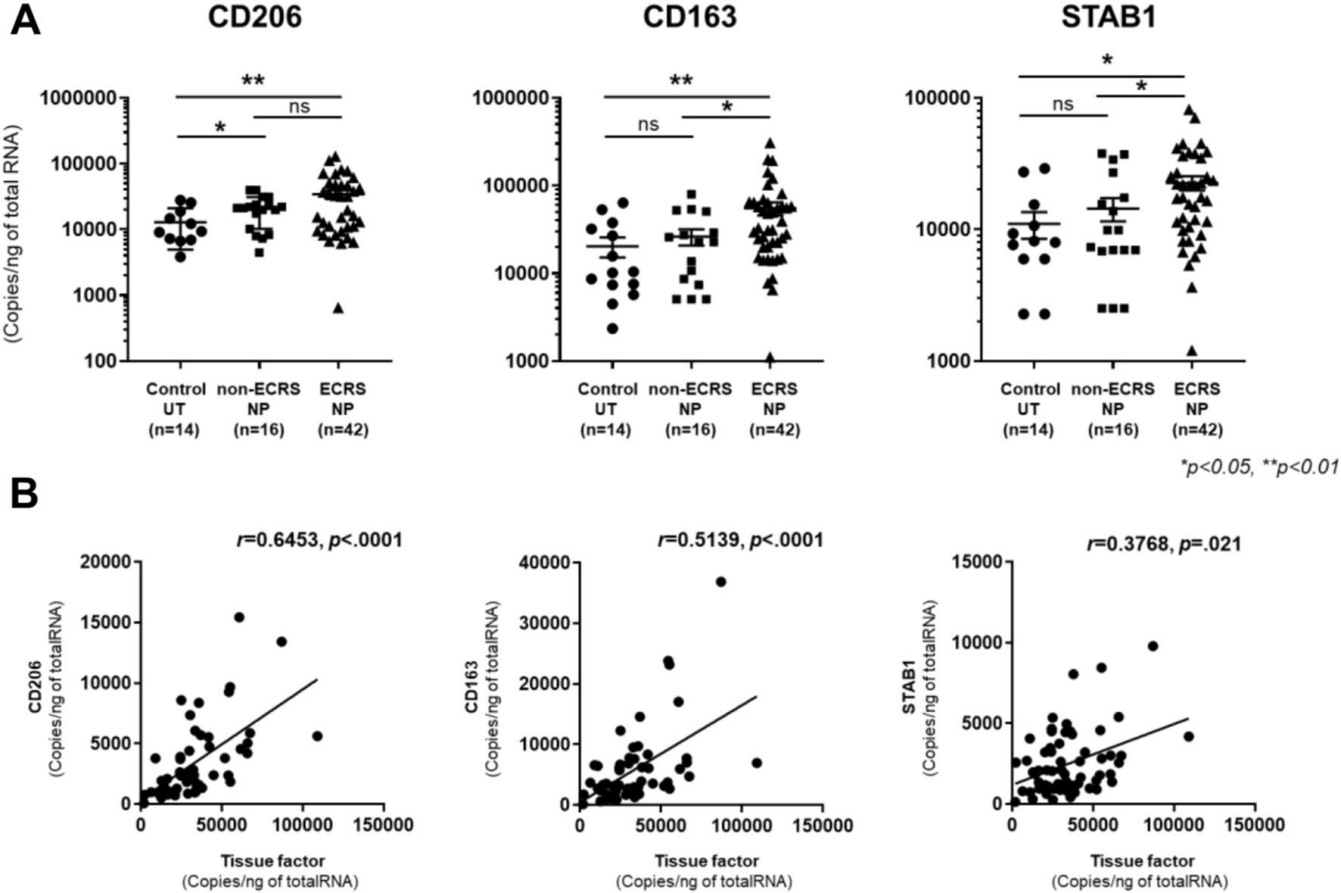
Correlation of TF with M2 macrophage markers in NP tissue. **A** Total RNA was extracted from UT of control participants (n = 14), NP tissues from patients with non-ECRS (n = 16), and NP tissues from patients with non-ECRS (n = 42). The expression of TF and M2 macrophage markers CD206, CD163, and STAB1 was analyzed using real-time PCR. **B** The correlation in NP tissue was assessed using the Spearman rank correlation test. *P < 0.05; **P < 0.01

**Fig. 6:**
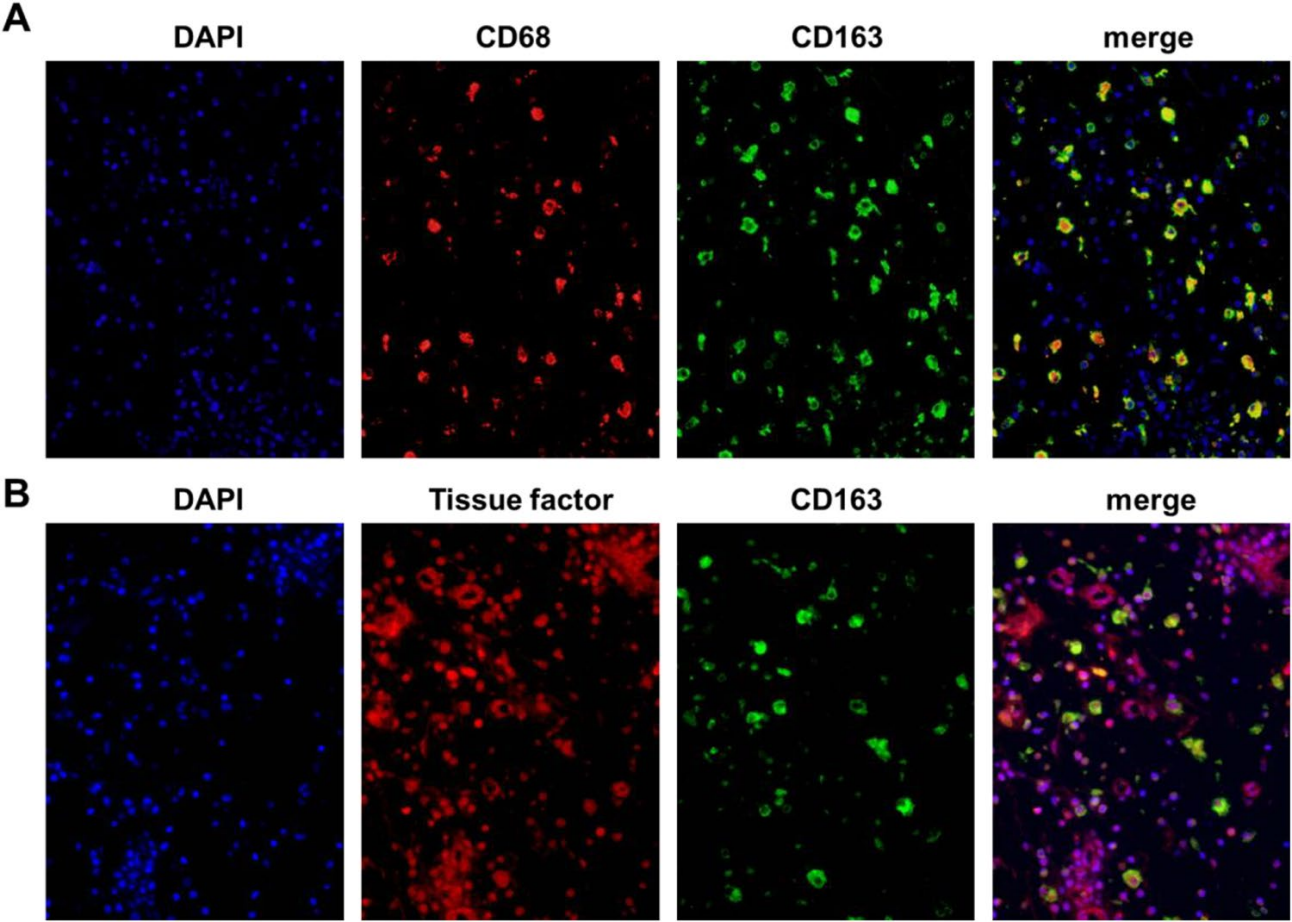
Detection of tissue facto (TF) in M2 macrophages in ECRS NP tissue. Immunofluorescence assay was performed with anti-CD68 mAb (red fluorescence) for macrophages, anti-CD163mAb (green fluorescence) for M2 macrophages, and nuclei were counterstained with DAPI (blue fluorescence) (**A**), and with anti-TF mAb, anti-CD163mAb (green fluorescence), and counterstained with DAPI (blue fluorescence) (**B**). The results are representative of four different individuals. DAPI, 4’,6-diamidino-2-phenylindole

Macrophages are versatile cells that can be polarized by the tissue environment. Classically activated macrophages (M1 macrophages) are induced by proinflammatory stimuli, such as Th1 cytokines (e.g., IFN-γ) or bacterial products (e.g., lipopolysaccharide). In contrast, alternatively activated macrophages (M2 macrophages) are induced by exposure to Th2 cytokines, including IL-4 and IL-13 [23]. Recent studies suggest that increased TF expression in macrophages contributes to inflammation-induced coagulopathy. Previously, we reported increased levels of M2 macrophages in NP tissues from patients with CRSwNP [12, 21, 24, 25]. Therefore, we examined whether M2 macrophages are primary TF-producing cells in ECRS NP tissue. First, we determined the levels of the M2 macrophage markers CD206, CD163, and stabilin 1 (STAB1) in the UT from control participants and NP tissue from patients with non-ECRS and ECRS. mRNA levels of CD206, CD163, and STAB1 were significantly elevated in ECRS NP tissues compared to UT from control (Fig. 5A). CD163 and STAB1 mRNA levels were also significantly higher in ECRS NP tissues than in non-ECRS NP tissues. Although CD206 tended to increase in ECRS NPs compared to non-ECRS NPs, this difference was not statistically significant (Fig. 5A). Additionally, TF expression significantly and positively correlated with CD206 (*r* = 0.6453, *P* < 0.0001), CD163 (*r* = 0.5139, *P* < 0.0001), and STAB1 (*r* = 0.3768, *P* = 0.021) expression (Fig. 5B). To further investigate whether M2 macrophages are the TF-producing cells in ECRS NPs, we performed immunofluorescence analysis using antibodies against macrophage marker CD68, M2 macrophage marker CD163, and an anti-TF antibody. Most CD68-positive cells were also positive for CD163 (Fig. 6A). Therefore, the infiltrated macrophages in ECRS NP tissues were M2 macrophages. Furthermore, a high degree of TF colocalization with CD163 was observed (Fig. 6B). These results suggest that M2 macrophages are an important source of TF in NP tissues of patients with ECRS.

### Type 2 cytokines and angiotensin II enhance TF expression in THP-1 cells

**Fig. 7:**
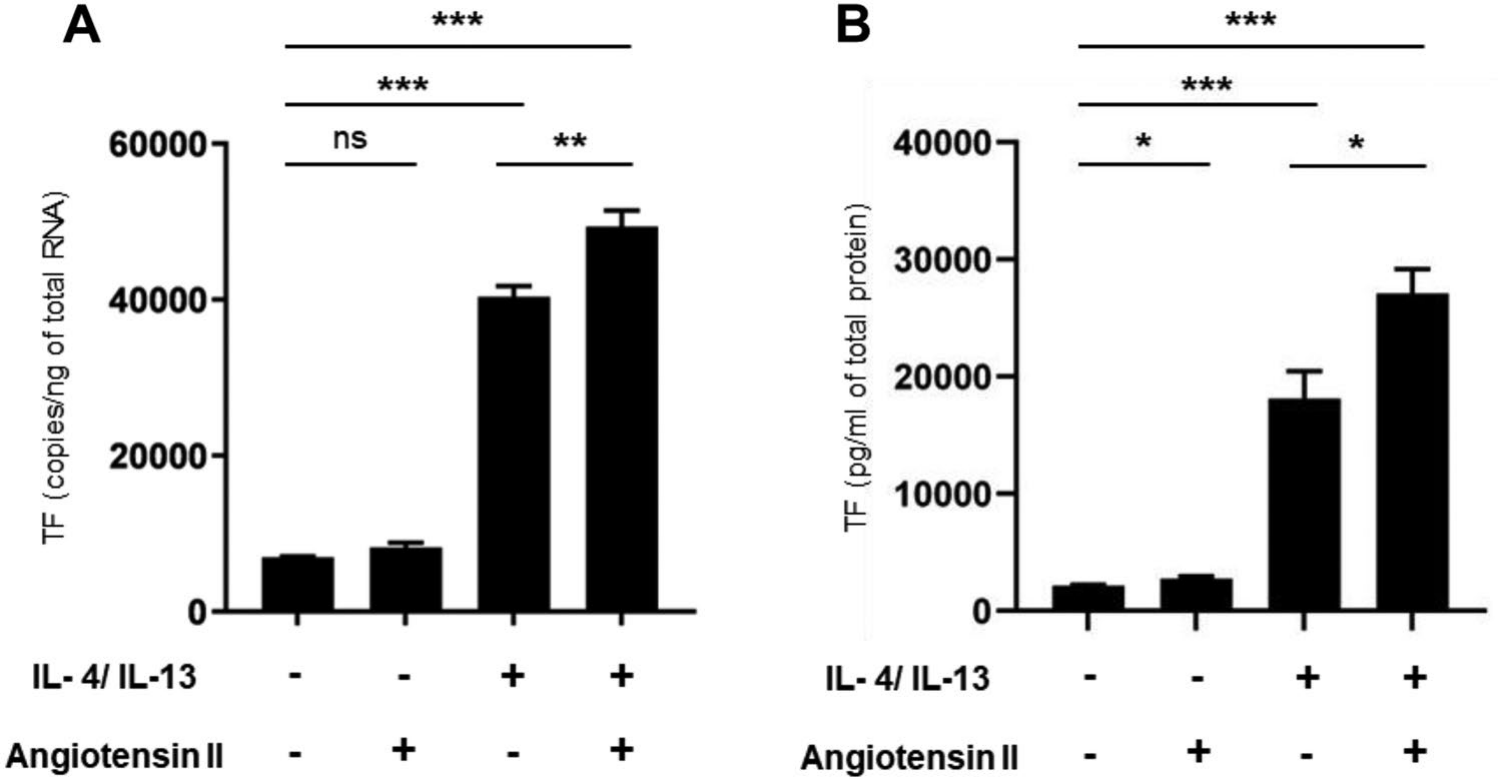
Regulation of tissue factor (TF) expression in macrophage. THP-1 cells were differentiated into macrophages using phorbol 12-myristate 13-acetate, then incubated with IL-4 and IL-13 to obtain M2 polarized macrophages. TF mRNA levels were determined by real-time PCR (**A**). TF protein concentrations in cell lysates from THP-1 cells were analyzed by ELISA. The TF concentration was normalized to the total protein concentration (**B**). Results are shown as the mean ± SEM of six independent experiments (n = 6). **P < 0.01; **P < 0.001

To study TF regulation in macrophages, human monocyte cell line THP-1 cells were used. THP-1 cells were differentiated into macrophages using phorbol 12-myristate 13-acetate, and then incubated with IL-4 and IL-13 to obtain M2 polarized macrophages as previously reported [26]. TF mRNA levels were significantly increased in M2 macrophages induced by type 2 cytokines and further increased with angiotensin II stimulation (Fig. 7A). To confirm at the protein level, TF concentrations in THP-1 cell lysates were measured using ELISA. Consistent with quantitative PCR data, the increased TF levels in M2 macrophages were further significantly increased by angiotensin II stimulation (Fig. 7B).

## Discussion

**Fig. 8:**
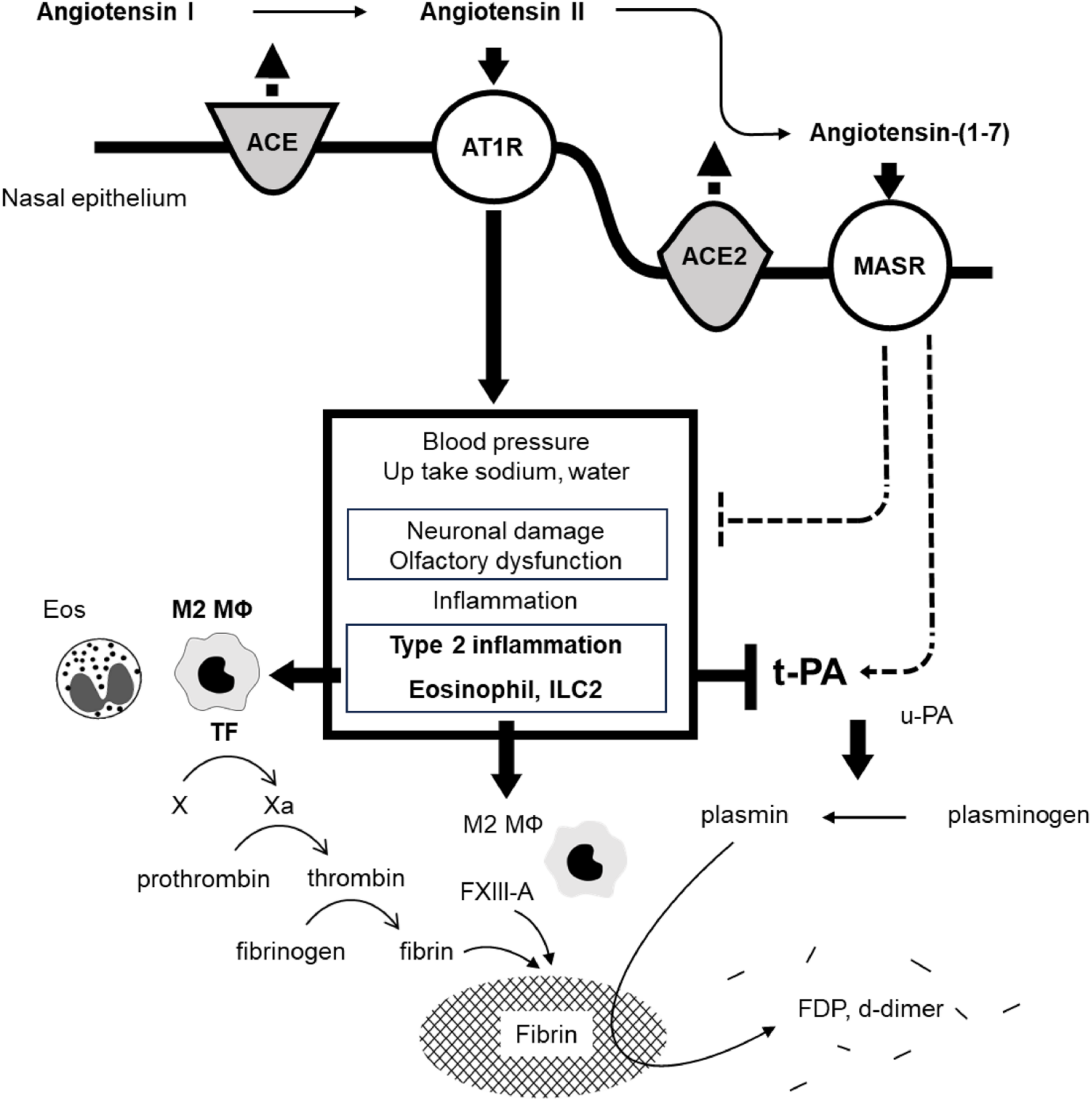
Hypothetical model of involvement of renin-angiotensin system (RAS) dysregulation in eosinophilic chronic rhinosinusitis (ECRS) pathogenesis. The ACE/angiotensin II/AT1R axis predominance over the ACE2/Ang-(1-7)/MASR axis might be involved in forming type 2 inflammatory milieu and subsequent skewing macrophage polarization toward M2 phenotype, resulting in increased tissue factor (TF) expression in macrophages. Upregulation of TF and FXIII-A in M2 macrophages plays a critical role in the formation of excessive fibrin deposition, facilitating NP development. In addition to type 2 inflammatory milieu, the ACE/angiotensin II/AT1R axis predominance may further reduce t-PA levels, causing impaired plasmin generation, which in turn, decreases fibrinolysis.

Systemic RAS is critical for regulating blood pressure and sodium and water balance. However, locally produced RAS have been attracting attention because they function independently of systemic RAS, play critical roles in tissue homeostasis, and are involved in tissue inflammation [22, 27, 28]. In this study, we assessed the expression of RAS components in the nasal mucosa of healthy control participants and in NP tissues from patients with non-ECRS and ECRS using real-time PCR, ELISA, and immunohistochemistry. ACE2 was primarily expressed in the nasal epithelium, and ACE2 levels were significantly decreased in NP tissues from patients with ECRS than those from patients with non-ECRS or UT from control participants (Fig. 1). In the RAS, ACE2 converts angiotensin II to Ang-(1–7), which counterbalances angiotensin II/AT1R signaling, exerting anti-inflammatory or anti-thrombotic effects (Fig. 8). We found an increasing trend in angiotensin II levels and significantly decreased Ang-(1–7) levels in ECRS NP tissues compared with non-ECRS NP tissues, likely due to reduced ACE2 expression (Fig. 2). Additionally, AT1R levels, the receptor for angiotensin II, were significantly increased, while MASR levels, the receptor for Ang-(1–7), were significantly decreased in ECRS NP tissues compared to non-ECRS NP tissues (Fig. 3). Collectively, these findings suggest that the ACE/angiotensin II/AT1R axis is more dominant than the ACE2/Ang-(1–7)/MASR axis in ECRS NP tissues and that RAS dysregulation may contribute to ECRS pathogenesis.

ACE2 also facilitates the cellular entry of SARS-CoV-2, and upregulating ACE2 in the airway epithelium has been suggested to be a potential risk factor for viral susceptibility and the severity of COVID-19 [29, 30]. Therefore, various studies have investigated ACE2 expression and distribution in the airway epithelium. Interestingly, ACE2 levels in the nasal mucosa are downregulated in type 2 airway inflammatory diseases, including allergic asthma, allergic rhinitis, and ECRS. Recent cohort studies of patients with COVID-19 demonstrated that coincident allergic airway diseases were associated with a significantly lower risk of hospitalization [13, 14, 31, 32]. However, to date, the role of RAS dysregulation, including decreased ACE2 levels, in ECRS pathogenesis has not been thoroughly explored.

RAS is the major hormonal system regulator of cardiovascular functions. More recently, RAS involvement in inflammatory responses across various organs has been unraveled [33]. Previous studies using asthmatic animal models showed that the angiotensin II/AT1R axis promotes type 2 cytokines production and eosinophil accumulation in lung tissue, whereas the Ang-(1–7)/MASR axis ameliorates allergic inflammation through counterregulatory mechanisms [27, 34–36]. The hallmarks of ECRS NP tissues are highly eosinophil infiltration linked to high levels of type 2 cytokines, including IL-4, IL-5, and IL-13, produced by various immune cells, such as Th2 cells, mast cells, and group 2 innate lymphoid cells (ILC2s), all of which are elevated in ECRS NP tissues [1]. Notably, ILC2 have recently garnered attention for their substantial production of type 2 cytokines in ECRS NP tissues. A recent study demonstrated that the angiotensin II/ AT1R axis enhances ILC2 responses in an allergic asthma mouse model [28]. This suggests that angiotensin II/AT1R axis predominance may contribute to establishing a type 2 inflammatory milieu in the ECRS nasal mucosa.

Recalcitrant NP tissues from patients with ECRS are important therapeutic targets due to symptoms such as nasal congestion, headache, and loss of smell. Histologically, NPs are characterized by intensely edematous stroma filled with plasma proteins, mainly albumin, exuded from capillaries by profound nasal or paranasal sinus mucosa inflammation.^1^ Reportedly, ACE2 downregulation and subsequent angiotensin II level elevation enhances vascular permeability [37]. Thus, the dominance of the angiotensin II/AT1R axis may contribute to NP tissue development.

Previously, we reported excessive fibrin deposition in NP tissues, which likely contributes to the retention of exuded plasma proteins from capillaries, thereby perpetuating mucosal edema and playing an etiological role in NP formation [9]. This study demonstrated significantly more intense fibrin deposition in ECRS NP tissues than in non-ECRS tissues (Fig. 4A, B). This increased fibrin deposition may explain the clinical observation that ECRS NP tissues are more recalcitrant to medical and surgical interventions and more likely to relapse than non-ECRS NP tissues [38]. We are now focusing on the dysregulation of the coagulation and fibrinolysis cascade that leads to excessive fibrin deposition in NP tissue. Activated eosinophils, which highly infiltrate NP tissues in patients with non-steroidal anti-inflammatory drugs-exacerbated respiratory disease (the most severe type of ECRS), express TF on the cell surface. Since TF initiates the extrinsic coagulation cascade, high TF levels in eosinophils may lead to profound fibrin deposition, resulting in NP development [12]. Additionally, we previously reported that coagulation factor XIII (FXIII)-A levels are increased in NP tissues from patients with CRSwNP and that M2 macrophages are the sole or major FXIII-A-producing cells in NPs [12]. FXIII-A participates in the final stage of the coagulation cascade, contributing to the strength and rigidity of the fibrin clot. The angiotensin II/AT1R axis reportedly promotes coagulation cascade by increasing TF expression in various tissues [39]. Hypercoagulopathy, a prominent complication of COVID-19, is caused by up-regulation of this axis [22]. As previously reported, although eosinophils expressed TF, unknown infiltrated cells also expressed TF in ECRS NP tissues [11]. In this study, M2 macrophages but not M1 macrophages, were significantly increased in ECRS NP tissues (Fig. 5). The increased presence of M2 macrophages could be explained by the aberrant type 2 inflammatory milieu in ECRS. Previous studies have demonstrated that the angiotensin II/AT1R axis promotes TF expression in various cells, including endothelial cells, vascular smooth muscle cells, and macrophages [21, 40, 41]. Our findings indicate that TF expression was significantly increased in M2 polarized macrophages compared to M0 or M1 macrophages and was further enhanced by angiotensin II stimulation but was unchanged by angiotensin II stimulation alone (Figs. 6 and 7). Collectively, these data support the hypothesis that angiotensin II/ AT1R axis dominance over the Ang-(1–7)/ MASR axis may contribute to type 2 inflammatory milieu and the subsequent skewing of macrophage polarization toward the M2 phenotype, resulting in increased TF expression in macrophages. Upregulating TF and FXIII-A in M2 macrophages play a critical role in excessive fibrin deposition, which may be involved in tissue remodeling in ECRS NP tissues (Fig. 8).

We previously found that NP tissues from patients with CRSwNP have significantly decreased tissue plasminogen activator (t-PA) levels, an important mediator of fibrinolysis, downregulated by type 2 cytokines in nasal epithelial cells, resulting in excessive fibrin deposition [9]. Reportedly, RAS plays a role in fibrinolysis, with angiotensin II/AT1R signaling reducing t-PA production, while inhibiting this pathway increases t-PA levels [42, 43]. Based on the current observation and previous reports, it is reasonable to speculate that the type 2 inflammatory milieu and RAS imbalance, particularly angiotensin II/ AT1R axis dominance, facilitate profound fibrin deposition in the nasal mucosa by accelerating coagulation cascade and impairing fibrinolysis, which in turn forms recalcitrant NP tissues in patients with ECRS (Fig. 8).

Olfactory dysfunction is an important clinical manifestation in patients with ECRS. However, its precise mechanisms beyond olfactory cleft constriction remain unclear. Notably, we have observed improvements in olfactory function immediately after dupilumab treatment, effectively suppressing IL-4 and IL-13 signaling in patients with ECRS. Therefore, it is likely that mechanisms other than preventing odor particles from reaching the olfactory epithelium by mucosal edema contribute to olfactory dysfunction in patients with ECRS. Recent studies have reported that angiotensin II signaling may be involved in neuronal damage, while its counter-regulator, Ang-(1–7) signaling, may be a promising therapeutic candidate through its neuroprotective effects [44, 45]. Interestingly, Marin et al. reported decreased ACE2 levels in the olfactory mucosa of patients with ECRS [46]. Thus, increased angiotensin II levels, caused by reduced ACE2 levels, might be implicated in olfactory disturbance in ECRS. Improving the RAS imbalance may be a promising therapeutic strategy for olfactory disturbance.

Several limitations should be acknowledged. Although our findings demonstrate an imbalance in RAS signaling associated with increased TF expression and fibrin deposition in ECRS, the study design is primarily observational and does not provide direct in vivo causal validation. While our in vitro experiments showed that angiotensin II enhanced TF expression in cultured macrophages under type 2 inflammatory conditions, interventional studies such as AT1R blockade would be required to establish definitive causality.

In addition, although TF expression was closely associated with infiltrating M2 macrophages, contributions from other inflammatory or structural cells cannot be excluded. Some parameters, including tissue angiotensin II levels, did not reach statistical significance, and a larger sample size may be necessary to confirm these findings. Furthermore, although systemic corticosteroids were discontinued at least two weeks prior to surgery, the potential influence of other concomitant medications on RAS components cannot be completely excluded.

In summary, we identified RAS imbalance and a predominance of the ACE/angiotensin II/AT1R axis over the ACE2/Ang-(1–7)/MASR axis in ECRS. This imbalance might be involved in disease pathogenesis. We also found significantly elevated TF levels in ECRS NP, primarily produced by M2 macrophages. Alongside the type 2 inflammatory milieu, the predominance of the ACE/angiotensin II/AT1R axis may facilitate skewing toward M2 macrophages and overproduction of TF, resulting in excessive fibrin deposition, which in turn forms NP tissue in patients with ECRS. These findings suggest that targeting local RAS dysregulation could offer therapeutic benefits for treating ECRS.

## Supplementary Information

Below is the link to the electronic supplementary material.

Supplementary file 1 (DOCX 50 KB).

Supplementary file 2 (XLSX 12 KB).

## Data Availability

No datasets were generated or analysed during the current study.
