# Supplementary material for: Renin–angiotensin imbalance promotes excessive fibrin deposition through M2 macrophage–derived tissue factor expression in eosinophilic chronic rhinosinusitis
Source: Inflamm Res. 2026 Apr 28;75(1):103. doi: 10.1007/s00011-026-02254-1 (PMC13124779; doi:10.1007/s00011-026-02254-1)
Supplement: Supplementary file 1 — (DOCX 50 KB). [file 11_2026_2254_MOESM1_ESM.docx]

**Online Data Supplement**

Renin–angiotensin imbalance promotes excessive fibrin deposition through M2 macrophage–derived tissue factor expression in eosinophilic chronic rhinosinusitis

Tetsuji Takabayashi, Kanako Yoshida, Yukinori Kato, Masafumi Sakashita, Shigeharu Fujieda

**Methods**

**Patients and sample preparation**

Patients diagnosed with chronic rhinosinusitis (CRS) were recruited from the Department of Otorhinolaryngology, Head and Neck Surgery of the University of Fukui. Nasal polyp (NP) tissues were obtained during routine functional endoscopic sinus surgery from patients with CRS with NP (CRSwNP). All patients met the diagnostic criteria for CRS as defined by the guidelines of the European position paper on rhinosinusitis and nasal polyps. Table E1 presents the participants’ characteristics. Patients with > 70 eosinophils per high-power field in their NP specimens were classified as having eosinophilic CRS (ECRS). Patients with an established immunodeficiency; pregnancy; coagulation disorder; or a diagnosis of classic allergic fungal sinusitis, Churg–Strauss syndrome, or cystic fibrosis were excluded from the study. Our study excluded patients treated with systemic or topical corticosteroids within 2 weeks before surgery. Besides corticosteroids, participants were on a variety of medications, including nonsteroidal anti-inflammatory drugs and antihistamines.

All participants provided informed consent, and the protocol and consent forms governing the study’s procedures were approved by the institutional review board of the University of Fukui, in accordance with the ethical principles contained in the Declaration of Helsinki.

**Real-time polymerase chain reaction (PCR)**

Nasal tissues were immediately placed in a stabilization reagent (RNAlater; Thermo Fisher Scientific, Waltham, MA, USA) and homogenized using a Multi-Beads Shocker (Yasui Kikai Corporation, Osaka, Japan) according to the manufacturer’s instructions. Total RNA was extracted using NucleoSpin RNA II (Macherey-Nagel, Bethlehem, PA, USA) with DNase I (Invitrogen, CA, USA) according to the manufacturer’s instructions. The quality of total RNA from the sinonasal tissues was assessed using a 2100 Bioanalyzer (Agilent Technologies, Santa Clara, CA, USA) with RNA 6000 Nano LabChip (Agilent Technologies). Single-stranded cDNA was synthesized using the High-Capacity cDNA Reverse Transcription Kit (Thermo Fisher Scientific). Semi-quantitative real-time RT-PCR was performed using the TaqMan method on an Applied Biosystems StepOnePlus Real-Time PCR system (Thermo Fisher Scientific) in 15 μl reactions (7.5 μl of 2× TaqMan Master mix [Thermo Fisher Scientific], 0.75 μl of 20× primer and probe mixture). Probes for ACE (Hs00174179_m1), ACE2 (Hs01085333_m1), AGTR1 (Hs05043708_s1), Mas (Hs00267157_s1), tissue factor (Hs01076029_m1), MMR (Hs00267207_m1), CD163 (Hs00174705_m1), STAB1 (Hs01109068_m1), Glyceraldehyde 3-phosphate dehydrogenase (GAPDH; Hs02786624_g1), and β-actin (ACTB; Hs01060665_g1) were purchased from Thermo Fisher Scientific. To determine the exact copy numbers of the target genes, aliquots of purified PCR fragments of the target genes were serially diluted and used as standards for each experiment. Aliquots of cDNA, equivalent to 10 ng of total RNA, were used for real-time PCR. The mRNA expression levels were normalized to the median expression of the housekeeping gene GAPDH or β-actin.

**Immunohistochemistry**

NP tissues and uncinate tissues (UT) were immediately fixed in 10% formalin, embedded in paraffin, and sectioned into 3-μm slices using a Retoratome REM-710 (Yamato Kohki, Saitama, Japan). Blocked sections were incubated overnight at 4 °C with mouse anti-human ACE2 mAb (66699-1-Ig; Proteintech, Rosemont, IL) at a 1:1000 dilution, mouse anti-human MAS1 mAB (sc-390453; Santa Cruz Biotechnology, Dallas, TX) at a 1:300 dilution, rabbit anti-human AGTR1/AT1R Ab (LS-C490145; Lynnwood, WA) at a dilution of 0.5 μg/ml, mouse anti-human fibrin mAb (SEKISUI diagnostics, Stamford, CT) at a 1:50 dilution, or rabbit anti-human tissue factor antibody (bs-4690R; Bioss Antibodies, Woburn, MA) at a 1:200 dilution. Equal concentrations of isotype control mouse IgG (ab37355; Abcam) were used for control experiments. After washing, the sections were incubated with ABC reagent (Vector Laboratories, Burlingame, CA, USA) for 1 h. The sections were rinsed, incubated in the DAB reagent (Invitrogen, Carlsbad, CA, USA), and counterstained with hematoxylin. Sections were dehydrated, cleaned, mounted, and cover-slipped using PARAmount-N (FALMA, Osaka, Japan). Microscopic analysis was performed using an Olympus BX53 upright research microscope with a 40× objective lens, and images were captured using CellSens software (Olympus, Tokyo, Japan). For the semi-quantitative analysis of ACE2, AT1R, MAS1, and fibrin, the slides were blinded, and 10 photographic fields were randomly taken from each slide and rated on a scale of 0 to 3 by a blinded observer. A rating of 0 indicated no staining, 1 indicated mild staining, 2 indicated moderate staining, and 3 indicated intense staining. The number of tissue factor-positive cells among infiltrating inflammatory cells was quantified by a blinded independent observer. Five randomly selected high-power fields (HPFs) per specimen were examined at ×400 magnification, and tissue factor-positive cells were manually counted. The mean value per case was used for statistical analysis. For immunofluorescence assay, rehydrated sections were blocked with blocking buffer (DAKO) and then incubated overnight at 4 °C with 24 ng/ml mouse anti-human CD68 mAb (clone PG-M1, IgG3, Thermo Fisher Scientific), 2.9 ng/ml mouse anti-human CD163 mAb (clone 10D6, IgG1, Thermo Fisher Scientific), and 1:200 dilution rabbit anti-human tissue factor antibody (bs-4690R; Bioss Antibodies, Woburn, MA). After washing, sections were incubated for 1 h at room temperature in the dark with 4 µg/mL Alexa Fluor 594-conjugated goat anti-mouse IgG3 (Invitrogen), 4 µg/mL Alexa Fluor 488-conjugated goat anti-mouse IgG1 (Invitrogen), 4 µg/mL Alexa Fluor 488-conjugated donkey anti-mouse IgG (Invitrogen), and 4 µg/mL Alexa Fluor 568-conjugated donkey anti-rabbit IgG (Invitrogen). Images from the immunofluorescence slides were captured with an Olympus IX71 inverted research microscope using a 40× objective lens, and images were processed using SlideBook software (Olympus).

**Enzyme-linked immunosorbent assay (ELISA)**

Freshly obtained tissue specimens were weighed, and 1 ml of phosphate-buffered saline supplemented with 0.05% Tween 20 (Sigma-Aldrich, St Louis, MO) and 1% protease inhibitor cocktail (Sigma-Aldrich) was added for every 100 mg of tissue. The tissue was then homogenized using a Multi-Beads Shocker (Yasui Kikai Corporation) according to the manufacturer’s instructions. After homogenization, the suspension was centrifuged at 4,000 rpm for 20 min at 4 °C, and supernatants were stored at −80 °C until analyzed. Protein concentrations of the tissue extracts and cell lysates were determined using a BCA Protein Assay Kit (Thermo Fisher Scientific). Before analysis, samples were thawed at room temperature and vortexed to ensure a well-mixed sample. Angiotensin II (Montigny-le-Bretonneux, France), angiotensin-(1-7) (Cloud-Clone Corp., Wuhan, China), and tissue factor (R&D Systems, Minneapolis, MN, USA) levels were assayed using specific ELISA kits according to the manufacturer’s instructions. Color intensity was measured using a Bio-Rad Spectrophotometer Model 680 Microplate Reader (Bio-Rad, Hercules, CA, USA) with associated software applied to the sandwich enzyme immunoassay technique. The concentrations of angiotensin II, angiotensin-(1-7), and tissue factor in the tissue homogenates and cell lysates were normalized to the total protein concentration.

**Cell culture**

The human monocytic THP-1 cells (Riken Cell Bank, Tsukuba Science City, Japan) were cultured in RPMI 1640 medium (Gibco Laboratories, Grand Island, NY, USA) supplemented with 10% defined fetal bovine serum (Gibco Laboratories) and 20 mM Hepes buffer (Gibco Laboratories) under humidified air with 5% CO_2_ at 37 °C. To differentiate THP-1 monocytes into macrophages, cells were treated with 100 nM phorbol 12-myristate 13-acetate (PMA, Sigma-Aldrich) for 24 h, followed by an additional 24 h incubation in RPMI medium. Macrophages were polarized into M2 macrophages by incubation with 20 ng/ml of IL-4 (R&D Systems) and 20 ng/ml of IL-13 (both from R&D Systems) for 72 h and then stimulated with 100 nM of angiotensin II (PEPTIDE INSTITUTE, Osaka, Japan) for 6 h.
